# Combined 5-Fluorouracil and Low Molecular Weight Heparin for the Prevention of Postoperative Proliferative Vitreoretinopathy in Patients With Retinal Detachment: A Meta-Analysis

**DOI:** 10.3389/fmed.2021.790460

**Published:** 2021-11-30

**Authors:** Chen Chen, Peng Chen, Xia Liu, Hua Li

**Affiliations:** ^1^Department of Ophthalmology, The Second People's Hospital of Yunnan Province (Affiliated Hospital of Yunnan University, Fourth Affiliated Hospital of Kunming Medical University), Kunming, China; ^2^Yunnan Clinical Medicine Center for Ocular Disease, Yunnan Eye Institute, Kunming, China; ^3^Key Laboratory of Yunnan Province for the Prevention and Treatment of Ophthalmic Diseases, Yunnan Eye Institute, Kunming, China; ^4^Department of Hepatopancreatobiliary Surgery, The Second Affiliated Hospital of Kunming Medical University, Kunming, China

**Keywords:** proliferative vitreoretinopathy, retinal detachment, vitrectomy, 5-fluorouracil, low molecular weight heparin, meta-analysis

## Abstract

**Background:** Postoperative proliferative vitreoretinopathy (PVR) remains a dilemma for retinal surgeons. We performed a literature search and meta-analyses to figure out whether combined 5-fluorouracil (5-FU) and low molecular weight heparin (LMWH) treatment were effective in improving the primary success of vitrectomy and preventing postoperative PVR occurrence in patients with retinal detachment (RD).

**Methods:** Databases including PubMed, Embase, the Cochrane library, and China National Knowledge Infrastructure (CNKI) were searched from inception to May 2021. Comparative studies approaching the effects of combined 5-FU and LMWH on postoperative PVR were included. Quality assessment was performed using RoB 2 and ROBINS-I tool. Study data were pooled using Review manager 5.4.1. The main outcomes were: the primary success of vitrectomy at 6 months and the postoperative PVR occurrence. The additional outcomes were: number of patients who underwent vitreoretinal reoperations and the number of vitreoretinal reoperations due to postoperative PVR. Subgroup analyses and sensitivity analyses were also performed.

**Results:** Six clinical trials with a total of 1,208 participants were included. We found that combined 5-FU and LMWH infusion did not improve the primary success of vitrectomy at 6 months (RR = 1.00, 95% CI = 0.95, 1.07, *P* = 0.89, *I*^2^ = 50%). Also, the conjunct therapy had no effect on reducing the number of patients who underwent vitreoretinal reoperations (RR = 1.00, 95% CI = 0.78, 1.28, *P* = 1.00, *I*^2^ = 42%). The overall effect of the treatment on preventing postoperative PVR was negative. However, in patients with PVR grade C (PVRC) before intervention, the 5-FU and LMWH treatment significantly reduced PVR occurrence. Visual acuity was not different between the treatment and control groups. Nevertheless, in one RCT, a significant reduction of VA was observed in the treatment group in macular-sparing patients with RD. No complications were attributed to the conjunct therapy.

**Conclusions:** The combined 5-FU and LMWH treatment neither improved the primary success of vitrectomy at 6 months nor decreased number of patients who underwent vitreoretinal reoperations. Thus, the treatment should not be routinely used in vitrectomy for patients with RD. However, the treatment proved beneficial in reducing postoperative PVR in patients with PVRC before intervention. More high-quality clinical trials are needed to confirm the results.

**Systematic Review Registration:** https://inplasy.com/inplasy-2021-8-0117/, identifier: INPLASY202180117.

## Introduction

Rhegmatogenous retinal detachment (RRD) is a common sight-threatening disease that has an incidence of 5–26.2 cases per 100,000 person-years (1, 2). Pars plana vitrectomy (PPV) is one of the principal treatments for RRD. The primary and final success rates for retinal reattachment reported for PPV were around 72.0 and 96.4%, respectively (3, 4). Proliferative vitreoretinopathy (PVR) is a common pathological process following the detachment of the retina and is reported to develop in 5–10% of all RRD (5, 6). PVR also occurs in eyes that underwent retinal reattachment surgery and is widely considered as one of the most common causes of failure of vitrectomy (1). PVR is characterized by the abnormal growth of epiretinal and/or subretinal membranes, which tend to contract and pull off the retina. The inflammation process, as well as the proliferation and migration of retinal pigment epithelium (RPE) cells, glial cells, and macrophages, are involved in the onset and development of PVR (6). Based on the understanding of these pathogeneses, various anti-inflammatory, and anti-metabolic drugs have been used in clinical trials to prevent the formation of postoperative PVR (7–9).

The antimetabolite 5-fluorouracil (5-FU) inhibits DNA synthesis as well as messenger RNA (mRNA) translation, thus it has been used in chemotherapy against tumors (10). In ophthalmic practice, 5-FU is selectively employed in trabeculectomy for reducing conjunctiva scarring (11). Kon et al. reported that single, short-term exposure to 5-FU significantly inhibited the proliferation of cultured human RPE cells (12). In experimental PVR models, 5-FU was sufficient in reducing vitreoretinal scarring and tractional retinal detachment (13). On the other hand, low molecular weight heparin (LMWH) has been proved to inhibit the proliferation of human scleral fibroblasts and RPE cells, as well as the cell-mediated traction of collagen gel *in vitro* (14). Moreover, LMWH reduced postoperative fibrin formation and proved beneficial in retinal repair in a group of patients with RD (15).

In 2001, Asaria et al. reported the combined use of 5-FU and LMWH in vitrectomy for the first time. They found that in patients at high risk of PVR, adjuvant therapy was effective in the prevention of postoperative PVR (16). However, later studies utilizing these two drugs presented controversial results in patients with RD with different severity (17, 18). In 2013, a Cochrane systematic review discussing the effects and safety of conjunct therapy was published. The authors discussed the topic on the basis of two RCTs without pooling the data, because of the high heterogeneity between the two trials (19). In the current study, we integrated results from relevant RCTs and non-randomized comparative studies, to further understand the efficacy and safety of combined 5-FU and LMWH treatment on the primary success of vitrectomy for RD as well as on the prevention of postoperative PVR.

## Materials and Methods

### Protocol and Registration

This meta-analysis was conducted and reported according to the guidelines of the Preferred Reporting Items for Systematic reviews and Meta-Analyses (PRISMA) (Supplementary data sheet 1) (20, 21). This study was registered at the International Platform of Registered Systematic Review and Meta-analysis Protocols (INPLASY) (registration number INPLASY202180117; https://inplasy.com/inplasy-2021-8-0117/).

### Eligibility Criteria

The participants, intervention, comparisons, outcomes, and type of studies (PICOS) of the current meta-analysis were as follows:

Participants (P): Patients over 16 years of age, diagnosed with RD, and scheduled for pars plana vitrectomy were included. Patients with traumatic retinal detachment or diabetic retinopathy were excluded.

Intervention (I): Infusion of combined 5-FU and LMWH during vitrectomy.

Comparisons (C): Placebo (normal saline) added to the infusion, or just normal infusion during vitrectomy with no additional drugs added.

Outcomes (O): The main outcomes include primary success at 6 months (defined as retinal reattachment after a single vitreoretinal operation) and postoperative PVR formation. The additional outcomes include the number of patients who underwent vitreoretinal reoperations, as well as the number of vitreoretinal reoperations due to postoperative PVR.

Type of studies (S): Prospective, controlled clinical trials were included, either randomized or non-randomized. Only studies that reported at least one of the main or additional outcomes and that had a follow-up period of no <6 months were included. Only studies written in English or Chinese were included. Retrospective studies and single-arm studies were excluded. Studies that had either 5-FU or LMWH for intervention, but not a combination of the two, were also excluded.

### Literature Search and Study Selection

We systematically searched the following electronic databases: PubMed, Embase, the Cochrane library, and China National Knowledge Infrastructure (CNKI), from inception to May 2021. We also searched the websites of ClinicalTrials.gov, WHO International Clinical Trials Registry Platform (ICTRP), and International Standard Randomized Controlled Trial Number (ISRCTN). We used a combination of the keywords to search, including “proliferative vitreoretinopathy,” “5-FU,” “5-fluorouracil,” “fluorouracil,” “low molecular weight heparin,” “dalteparin,” “enoxaparin,” “nadroparin,” and “tinzaparin.” Our search strategy was described in detail in Supplementary data sheet 2. Records were imported to the Endnote software (Clarivate Analytics, Philadelphia, USA), and duplicates were removed. Subsequently, the titles and abstracts of the records were screened, and irrelevant records were removed. The full texts of the remaining records were retrieved and judged according to our inclusion criteria. Two reviewers (CC and PC) independently performed the literature search and records screening, any disagreements were solved by discussion, or by consulting a third reviewer (HL).

### Quality Assessment and Data Extraction

For the included RCTs, we assessed the study quality with the updated Cochrane risk-of-bias tool (RoB 2) (22). Bias was evaluated in five domains (the randomization process, deviations from intended interventions, missing outcome data, measurement of the outcome, and selection of the reported result) according to the instructions of the tool (22). For non-randomized studies of the effects of interventions (NRSI), the Risk Of Bias In Non-randomized Studies—of Interventions (ROBINS-I) tool was utilized to assess the bias (23). After quality assessment, we extracted the following data from each included study: first author, year of publication, country where the study was carried out, language, study design, participants, mean age, treatment groups, number of eyes, 5-FU and LMWH infusion time, follow-up period, and intraocular tamponade. We also extracted the ratio of patients with preoperative PVR grade C (PVRC) or calculated the ratio from the original data. Two reviewers (CC and PC) independently assessed the study quality and extracted the data, consensus was reached by discussion or by consulting a third reviewer (HL).

### Data Analysis and Synthesis

Review manager 5.4.1 (Revman, Cochrane Collaboration, Oxford, UK) was used to analyze and synthesize the study data. The number of patients with events and the total number of patients from the treatment and control group were input, and the risk ratio (RR) was calculated automatically, along with its 95% CI. We performed tests for heterogeneity before data synthesis. If *I*^2^ ≤ 50%, heterogeneity was judged to be low or moderate, and fixed-effect model was used to pool the data. If *I*^2^ > 50%, heterogeneity was judged to be substantial, and a random effect model was used to synthesize the data. For the treatment effect, *P* < 0.05 was considered to be statistically significant.

For each outcome, we performed subgroup analyses to approach the origin of heterogeneity, as well as the possible differential effects of the 5-FU and LWMH treatment among the subgroups. The subgroups were divided according to the severity of the disease (different preoperative PVRC ratio), patient age, 5-FU and LMWH infusion time, and inclusion or exclusion of patients who underwent the previous vitrectomy.

Sensitivity analysis was performed by sequentially omitting individual studies, to assess the robustness of the current meta-analysis. If *I*^2^ decreased substantially, the omitted study would be considered as the source of heterogeneity. If the final effect changed after a specific study was omitted, for example, *P*-value changed from >0.05 to <0.05, the meta-analysis for this outcome would be considered to be unstable.

## Results

### Study Characteristics

**Figure 1:**
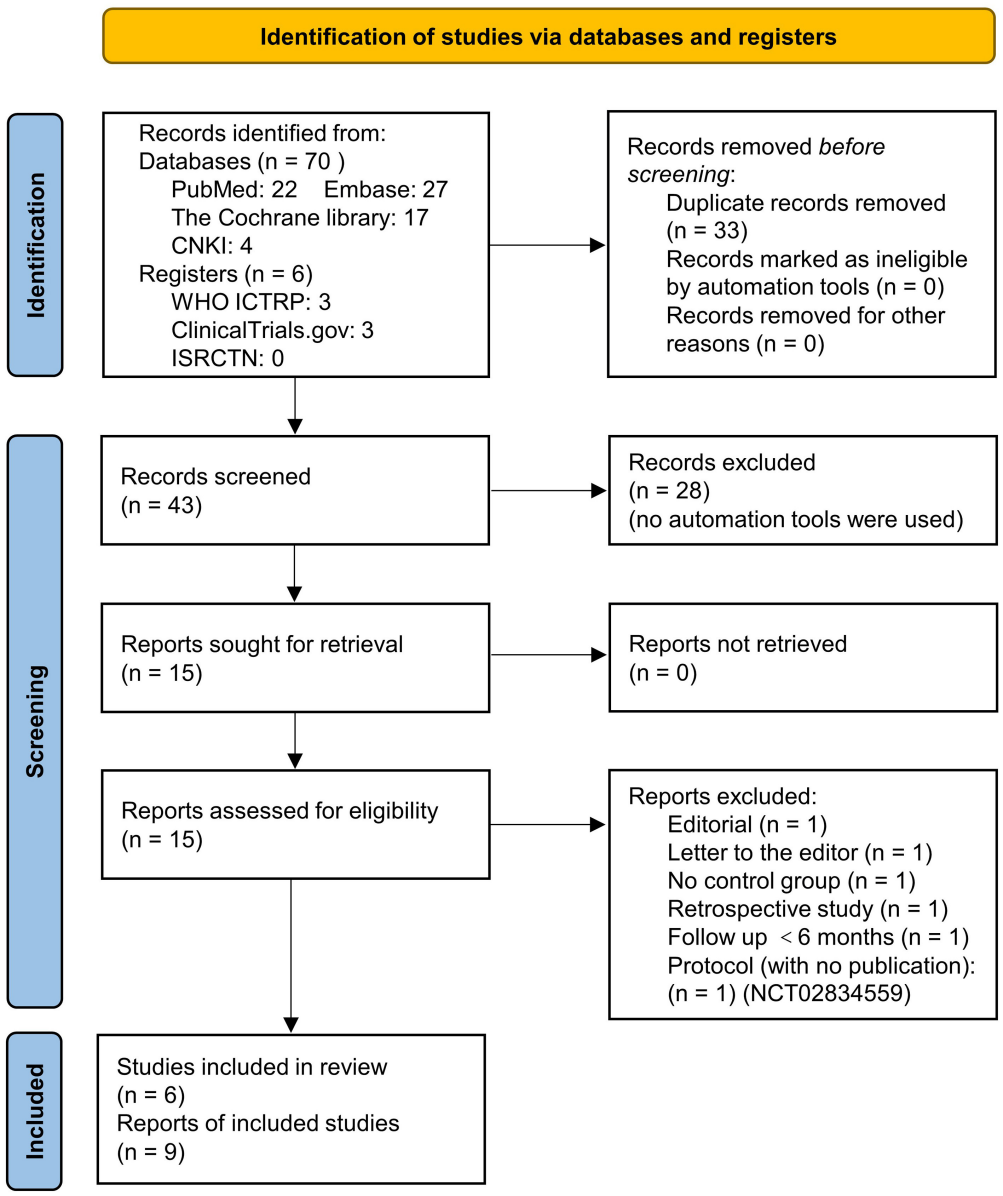
Preferred Reporting Items for Systematic reviews and Meta-Analyses (PRISMA) 2020 flow diagram of literature search and screening.

In our comprehensive literature search, 76 records were identified. After duplicates and irrelevant records removal, 15 full-text publications were screened and six were excluded with reasons, as indicated in the PRISMA flow diagram (Figure 1). Finally, six studies (nine reports) (16–18, 24–29) with a total of 1,208 patients were included in the current meta-analysis. These studies were conducted in the UK (16–18), India (24), China (29), and Venezuela (25), and were published between 2001 and 2014 (Table 1). Among them, five studies were published in English and one in Chinese. There were four randomized controlled trials (RCTs) and two non-randomized studies of the effects of interventions (NRSI). Two were multicenter studies (17, 18) and four were conducted in a single center (16, 24, 25, 29). In all six studies, the infusion concentration of 5-FU and LMWH was 200 μg/ml and 5 IU/ml, respectively. The control group received a placebo (normal saline) added to the infusion in three studies (16, 17, 24), and no drugs were added (just normal infusion) in the other three studies (18, 25, 29). Concerning the participants, one study enrolled unselected RRD patients with the preoperative PVRC rate of 1.8% in the treatment group and 3.7% in the control group (18), two studies enrolled RRD patients at high risk of postoperative PVR with the preoperative PVRC rate ranging from 13.8 to 70% (16, 24), three studies enrolled RD patients with 100% PVRC (17, 25, 29) (Table 1). Preoperative PVR was graded according to the updated classification by the Retina Society (1991) (30) in five studies (16–18, 24, 25), and was graded according to the 1983 classification (31) in one study (29). In the two editions of classifications, grades A and B were the same. The 1983 classification included grade C and D, and the 1991 updated classification included only grade C. We combined preoperative PVR grade C and grade D from Zhu (2006) (29) to be PVRC, in order to keep consistency. The infusion time of 5-FU and LMWH was no more than 60 min in three studies (17, 18, 29), while in the other three studies, the infusion lasted until air exchange (16, 24, 25). The average age of patients were <60 years in three studies (24, 25, 29), and more than 60 years in other three studies (16–18). In two studies, patients scheduled for primary vitrectomy were included (16, 18), while in the other four studies, a proportion of participants had undergone previous vitrectomy (17, 24, 25, 29).

**Table 1:** Characteristics of included studies.

**References**	**Year**	**Location**	**Language**	**Design**	**Patients**	**Preoperative PVRC ratio** **(Treatment/Ctrl)**	**Group**	**5-FU+LMWH infusion time**	**Number of eyes** **(Treatment/Ctrl)**	**Mean age (years)** **(Treatment/Ctrl)**	**Follow-up period**	**Tamponade**
Asaria et al. (16)	2001	UK	English	RCT	RRD patients at high risk of PVR	13.8/17.2%	5-FU+LMWH vs. Control	Infusion lasts until air exchange	87/87	62/64.3	6 m	SF_6_, C_3_F_8_, Silicone oil
Charteris et al. (17)	2004	UK	English	RCT	RD patients with PVRC	100/100%	5-FU+LMWH vs. Control	≤ 60 min	73/84	65.8/66.2	12 m	Silicone oil
Wickham et al. (18)	2007	UK	English	RCT	RRD patients (unselected)	1.8/3.7%	5-FU+LMWH vs. Control	≤ 60 min	342/299	61.9/61.4	6 m	SF_6_, C_3_F_8_, Silicone oil
Ganekal and Dorairaj (24)	2014	India	English	RCT	RRD patients at high risk of PVR	70/60%	5-FU+LMWH vs. Control	Infusion lasts until air exchange	20/20	28.5/38.5	6 m	Silicone oil, C_3_F_8_, Air
Zhu et al. (29)	2006	China	Chinese	NRSI	RD patients with PVRC	100/100%	5-FU+LMWH vs. Control	≤ 60min	66/66	46.2/45.4	6 m	C_3_F_8_, Silicone oil
Garcia et al. (25)	2007	Venezuela	English	NRSI	RD patients with PVRC	100%/100%	5-FU+LMWH vs. Control	Infusion lasts until air exchange	33/31	55/56.7	12 m	Silicone oil

### Risk of Bias Assessment

**Figure 2:**
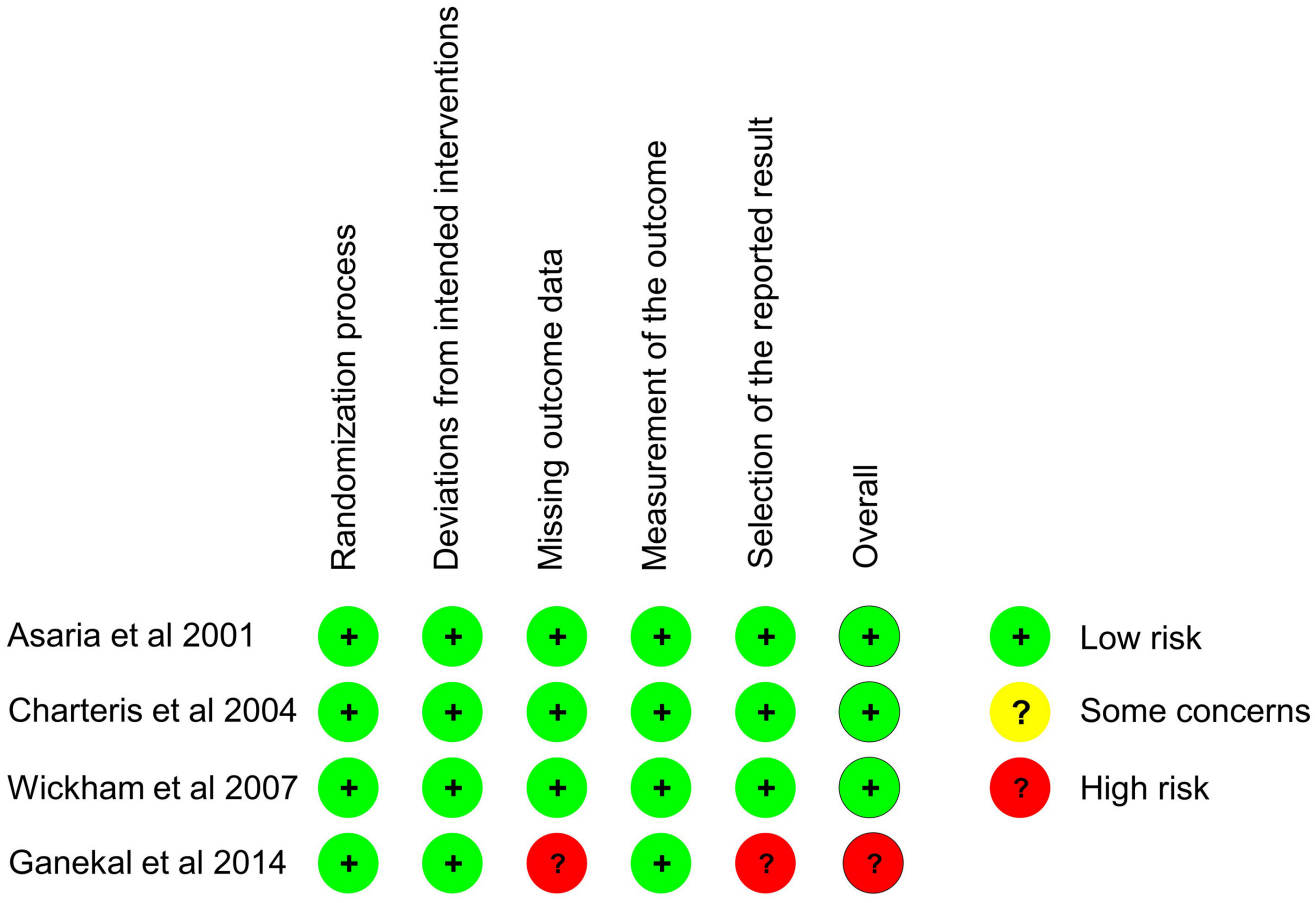
Summary of risk of bias assessment for RCTs using RoB 2.

The baseline characteristics of participants in the treatment and control groups were comparable in all six studies. All four RCTs (16–18, 24) performed concealment in allocation and during operation, three of them (16–18) mentioned the blinding of the outcome measurement personnel. Using the RoB 2 tool, three of the four RCTs were judged to be at low risk of bias across all five domains (16–18). However, one RCT was judged to be at high risk of bias because of obvious data missing and possible selection of the reported result (24) (Figure 2). For the two NRSI (25, 29), both studies were assessed to be at low or moderate risk of bias across all seven domains, thus, they were judged to be at an overall moderate risk of bias by the ROBINS-I tool (Table 2).

**Table 2:** Quality assessment of non-randomized comparative studies using ROBINS-I tool.

				**ROBINS-I domains**				
**Study**	**Bias due to confounding**	**Bias in selection of participants into the study**	**Bias in classification of interventions**	**Bias due to deviations from intended interventions**	**Bias due to missing data**	**Bias in measurement** **of outcomes**	**Bias in selection of the reported result**	**Overall ROBINS-I judgment**
Zhu et al. (29)	Moderate	Low	Moderate	Low	Low	Moderate	Moderate	Moderate
Garcia et al. (25)	Moderate	Low	Low	Low	Low	Low	Moderate	Moderate

### Main Outcomes

#### Primary Success at 6 Months

**Figure 3:**
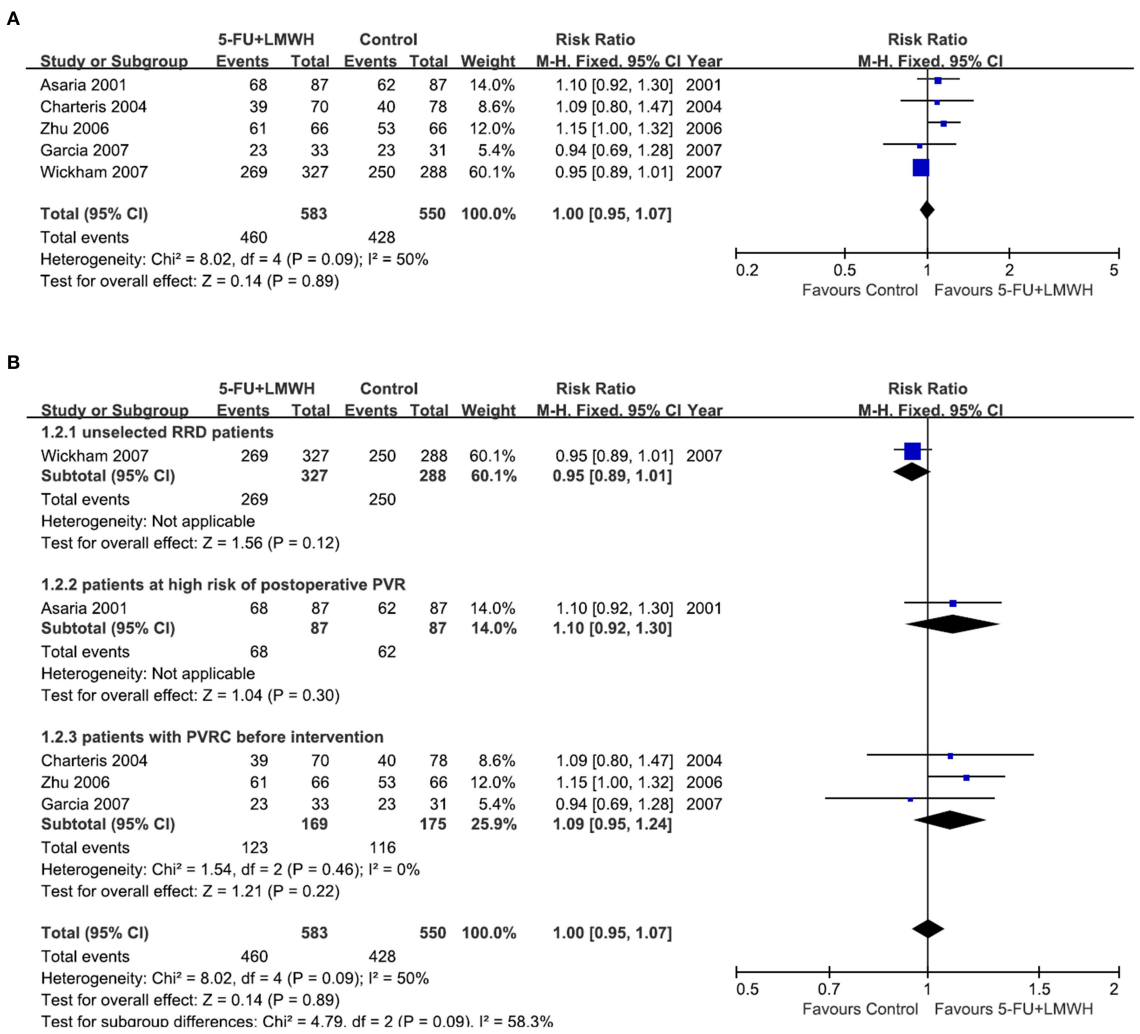
Forest plots for the meta-analysis of primary success at 6 months. **(A)** Overall meta-analysis; **(B)** Subgroup analysis according to preoperative PVRC ratio.

Stable retinal reattachment is the standard for judging the success of vitrectomy for RRD. In our meta-analysis, data from five studies were pooled to analyze this outcome (16–18, 25, 29). The primary success number was either reported in the study (16–18), or calculated from the retinal redetachment ratio (29), or counted from the original data table (25). The pooled data demonstrated that 5-FU and LMWH infusion did not improve the primary success of vitrectomy at 6 months (RR = 1.00, 95% CI = 0.95, 1.07, *P* = 0.89, *I*^2^ = 50%) (Figure 3A).

Then we performed subgroup analyses to determine the origin of heterogeneity. When we defined subgroups according to different preoperative PVRC ratios, the heterogeneity decreased to 0%, indicating that the difference in preoperative PVRC ratio among the studies may be the primary source of heterogeneity. The *P*-values of the overall effect in all three subgroups were >0.05, indicating that the 5-FU and LMWH infusion improved the primary success in none of the subgroups (Figure 3B). We also defined the subgroups according to 5-FU and LMWH infusion time (≤ 60 min or lasted until air exchange), the average age of participants (≤ 60 years or >60 years), and inclusion or exclusion of patients who underwent the previous vitrectomy. We found that these factors were not the primary source of heterogeneity, as *I*^2^ did not decrease substantially. Also, the overall effect of 5-FU and LMWH was not significant in any subgroup, as indicated by the *P* values of > 0.05 (Supplementary Figure 1).

To evaluate the robustness of the meta-analysis for this outcome, we performed sensitivity analysis by sequentially omitting individual studies. We found that Zhu (2006) (29) and Wickham (2007) (18) were the primary sources of heterogeneity since the *I*^2^ decreased substantially after either of the two studies was omitted (Table 3). Nevertheless, the overall effect of 5-FU and LMWH was not changed (*P*-value remained > 0.05), indicating that our meta-analysis for this outcome was stable.

**Table 3:** Sensitivity analysis for main outcome: primary success at 6 months.

**Omitted study**	**RR**	**95% CI**		**P of chi-square**	***I*** **^2^**	**Overall effect**	**Selected model**
None	1	0.95	1.07	0.09	50%	*P* = 0.89	Fixed-effect model
None	1.04	0.93	1.15	0.09	50%	*P* = 0.51	Random-effect model
Asaria et al. (16)	1.02	0.9	1.16	0.08	55%	*P* = 0.72	Random-effect model
Charteris et al. (17)	1.03	0.92	1.16	0.05	61%	*P* = 0.59	Random-effect model
Zhu et al. (29)	0.98	0.92	1.05	0.36	7%*	*P* = 0.63	Fixed-effect model
Garcia et al. (25)	1.05	0.93	1.18	0.05	62%	*P* = 0.42	Random-effect model
Wickham et al. (18)	1.09	0.98	1.21	0.68	0%*	*P* = 0.11	Fixed-effect model

* *Heterogeneity decreased after a particular study was omitted*.

#### Postoperative PVR Occurrence

**Figure 4:**
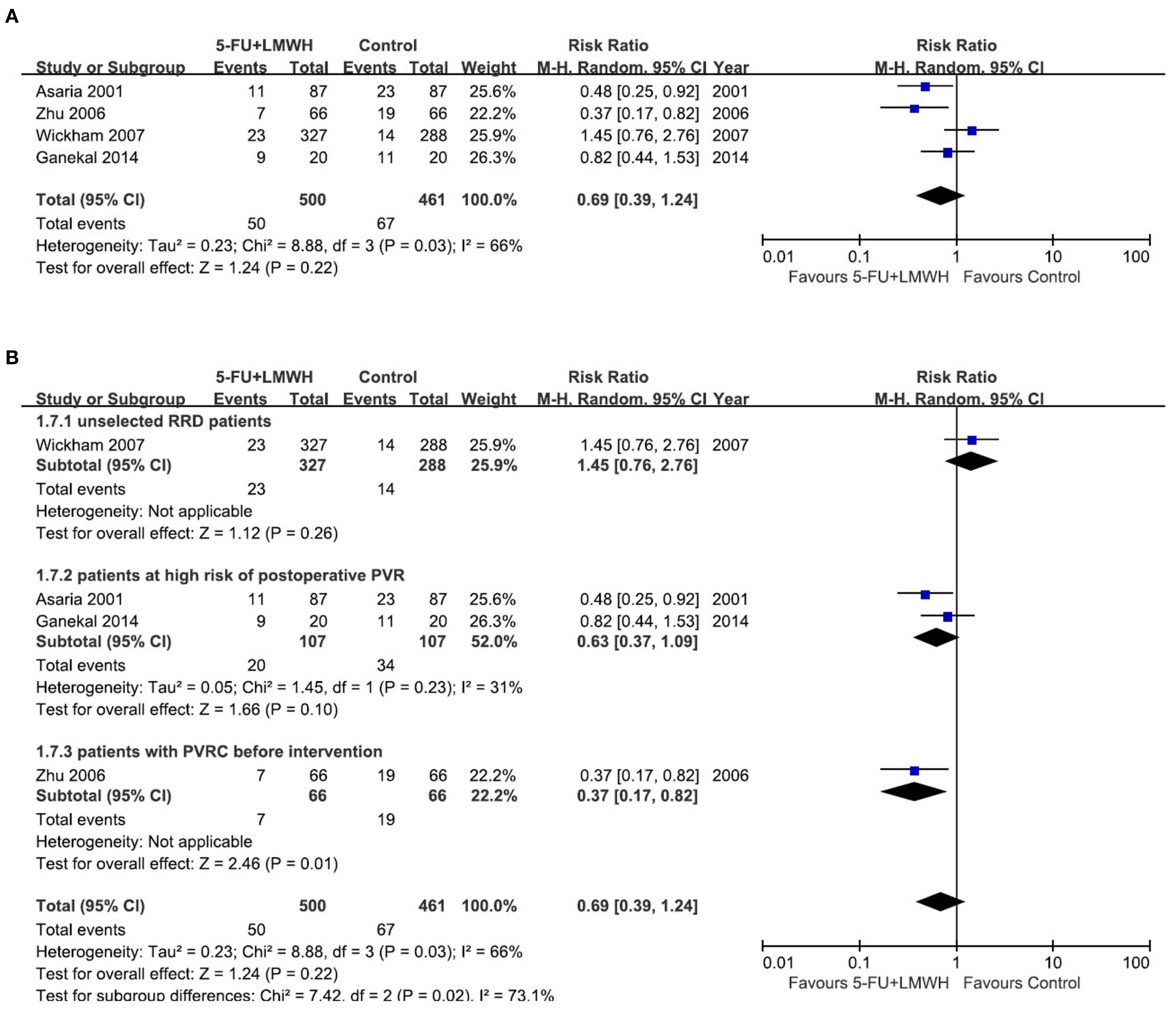
Forest plots for the meta-analysis of postoperative proliferative vitreoretinopathy (PVR) occurrence. **(A)** Overall meta-analysis; **(B)** Subgroup analysis according to the preoperative PVRC ratio.

**Figure 5:**
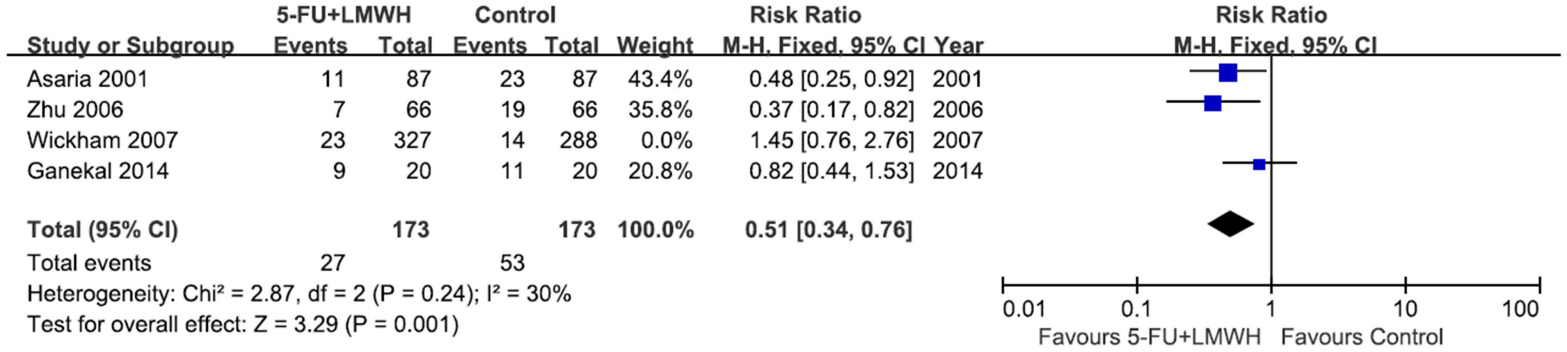
Forest plot for the meta-analysis of postoperative PVR occurrence when the study Wickham 2007 was omitted.

Postoperative PVR occurrence was reported in four studies (16, 18, 24, 29). The pooled data indicated the ineffectiveness of the combined 5-FU and LMWH in preventing postoperative PVR (RR = 0.69, 95% CI = 0.39, 1.24, *P* = 0.22, *I*^2^ = 66%) (Figure 4A). As *I*^2^ > 50%, we considered significant heterogeneity, and subgroup analyses were carried out. The effect of 5-FU and LMWH on preventing PVR was significant in the subgroup of patients with PVRC before intervention (Figure 4B). However, this subgroup contained only one study (29). Subgroup analyses defined by other factors did not show any significant change in the effect of 5-FU and LMWH or heterogeneity (Supplementary Figure 2). In the sensitivity analysis for this outcome, we found that when Wickham (2007) (18) was omitted, the heterogeneity decreased (*I*^2^ changed from 66 to 30%), and the overall effect of the combined 5-FU and LMWH changed. A significant effect against postoperative PVR formation was shown, as indicated by the *P*-value of 0.001 (Table 4, Figure 5).

**Table 4:** Sensitivity analysis for main outcome: postoperative PVR occurrence.

**Omitted study**	**RR**	**95% CI**		**P of chi-square**	***I*** **^2^**	**Overall effect**	**Selected model**
None	0.69	0.39	1.24	0.03	66%	*P* = 0.22	Random-effect model
Asaria et al. (16)	0.78	0.38	1.63	0.03	71%	*P* = 0.51	Random-effect model
Zhu et al. (29)	0.83	0.45	1.54	0.06	64%	*P* = 0.55	Random-effect model
Wickham et al. (18)	0.51	0.34	0.76	0.24	30%*	*P* = 0.001**	Fixed-effect model
Ganekal and Dorairaj (24)	0.65	0.28	1.49	0.01	77%	*P* = 0.31	Random-effect model

* *Heterogeneity decreased after a particular study was omitted*.

** *Overall effect changed when a particular study was omitted*.

### Additional Outcomes

#### Number of Patients Who Underwent Vitreoretinal Reoperations

**Figure 6:**
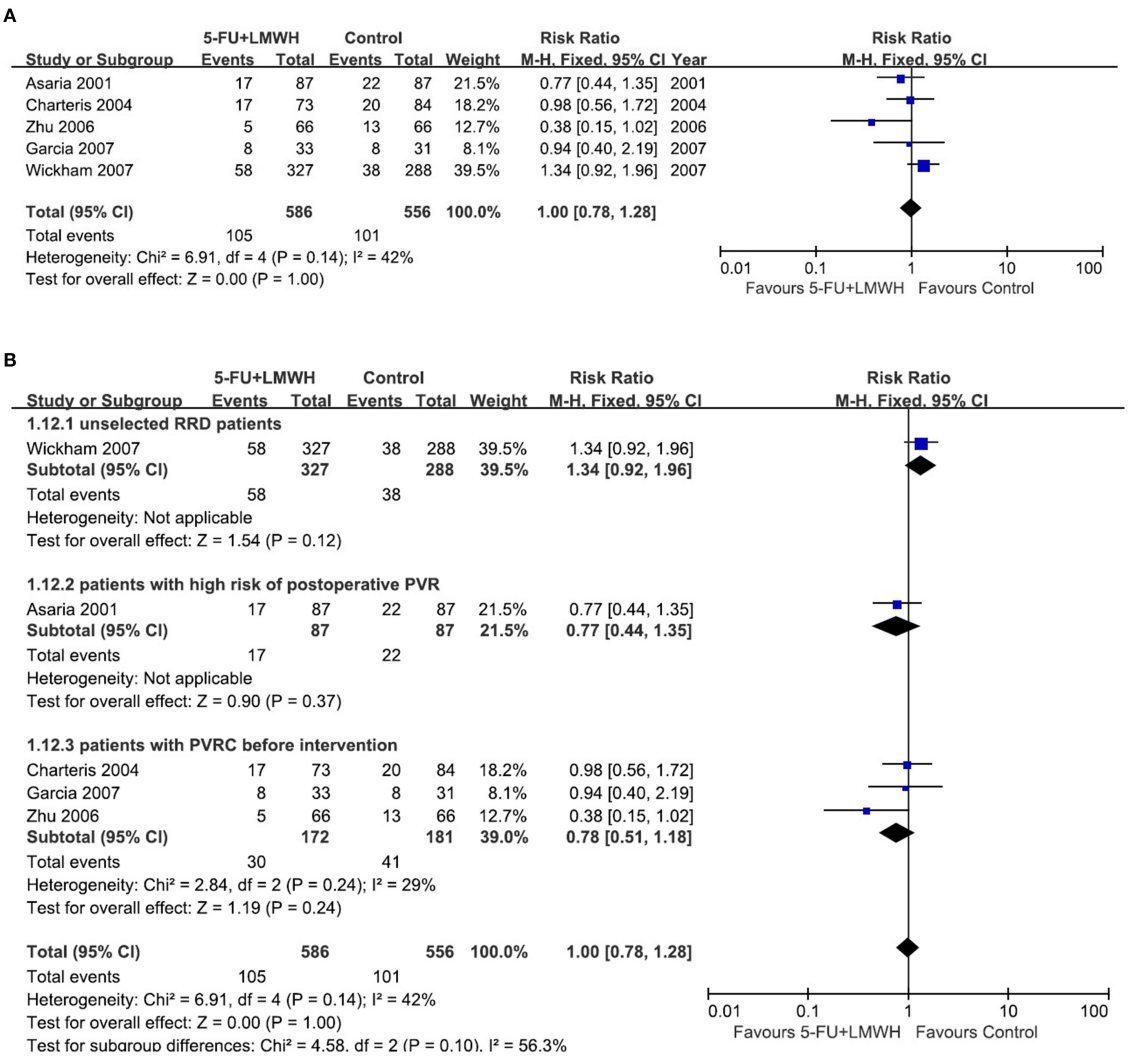
Forest plots for the meta-analysis of the number of patients who underwent vitreoretinal reoperations. **(A)** Overall meta-analysis; **(B)** Subgroup analysis according to preoperative PVRC ratio.

Reoperation is a remedy for failed primary vitrectomy. However, surgery *per se*, will stimulate the tissue repairing and may contribute to PVR formation. Our meta-analysis for this outcome pooled the data from five studies (16–18, 25, 29). We found that the combined 5-FU and LMWH therapy was ineffective in reducing the number of patients who underwent vitreoretinal reoperations (RR = 1.00, 95% CI = 0.78, 1.28, *P* = 1.00, *I*^2^ = 42%) (Figure 6A). The subgroup analysis according to the preoperative PVRC level decreased the *I*^2^ from 42 to 29%, however, the effect of 5-FU and LMWH was not significantly changed in any subgroup (Figure 6B). 5-FU and LMWH infusion time seemed to be the source of heterogeneity during the meta-analysis for this outcome since the *I*^2^ decreased dramatically in subgroups divided by this factor, while age and inclusion/exclusion of patients who underwent the previous vitrectomy did not influence the heterogeneity (Supplementary Figure 3). Nevertheless, the effect of 5-FU and LMWH was not significantly changed in any subgroup analysis (Supplementary Figure 3).

By sensitivity analysis, we found Zhu (2006) (29) and Wickham (2007) (18) were the sources of heterogeneity since the omission of either study drastically decreased the heterogeneity (*I*^2^ changed to 0%) (Table 5). However, the overall effect of 5-FU and LMWH in reducing the number of patients who underwent vitreoretinal reoperations was not changed, indicating that our meta-analysis for this outcome was quite stable.

**Table 5:** Sensitivity analysis for additional outcome: number of patients underwent vitreoretinal reoperations.

**Omitted study**	**RR**	**95% CI**		**P of chi-square**	***I*** **^2^**	**Overall effect**	**Selected model**
None	1	0.78	1.28	0.14	42%	*P* = 1.00	Fixed-effect model
Asaria et al. (16)	1.06	0.8	1.4	0.12	49%	*P* = 0.67	Fixed-effect model
Charteris et al. (17)	0.88	0.54	1.42	0.08	57%	*P* = 0.59	Random-effect model
Zhu et al. (29)	1.09	0.84	1.41	0.41	0%*	*P* = 0.52	Fixed-effect model
Garcia et al. (25)	0.9	0.58	1.39	0.08	56%	*P* = 0.64	Random-effect model
Wickham et al. (18)	0.78	0.55	1.08	0.42	0%*	*P* = 0.14	Fixed-effect model

* *Heterogeneity decreased after a particular study was omitted*.

#### Number of Vitreoretinal Reoperations Due to Postoperative PVR

**Figure 7:**
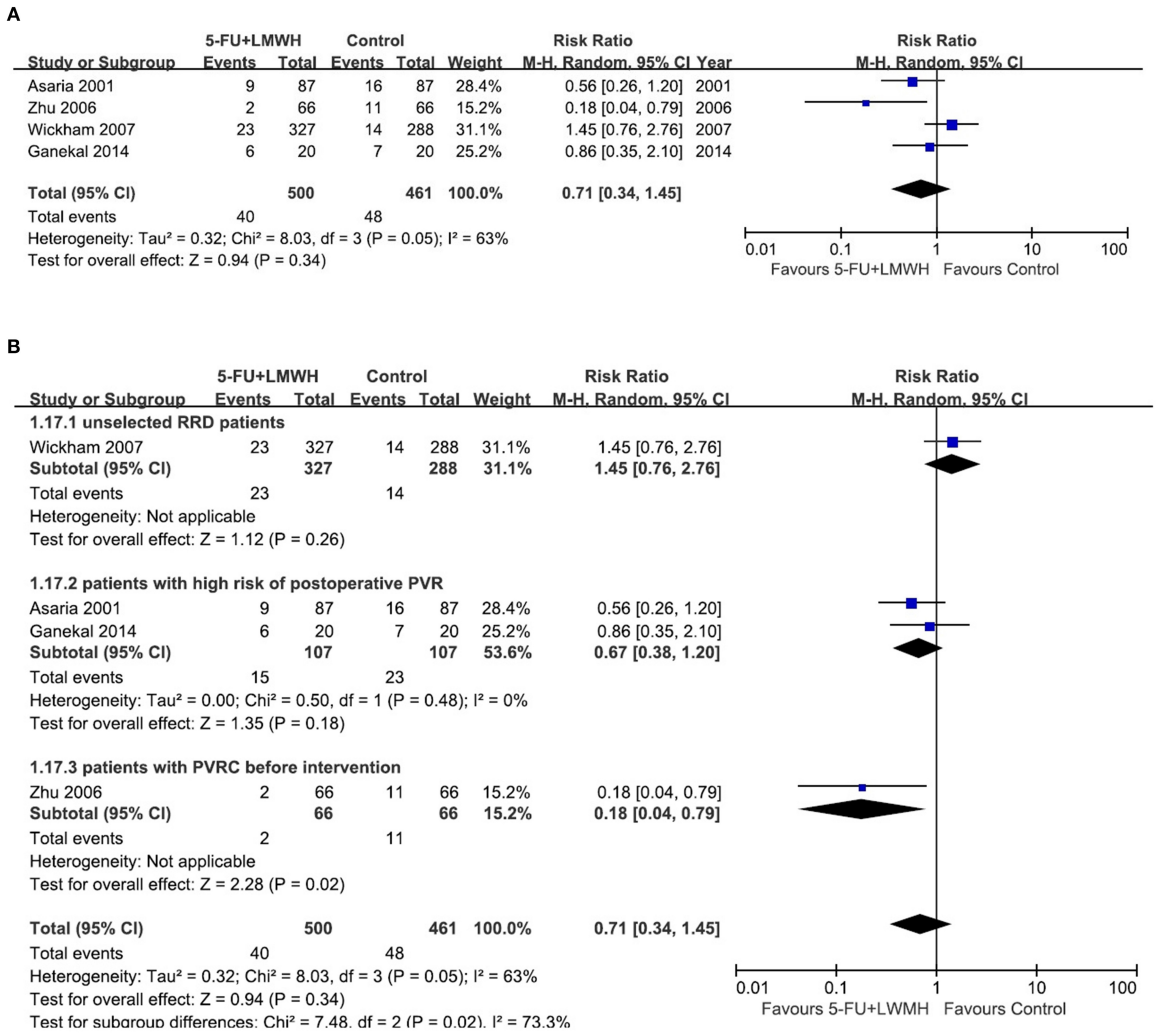
Forest plots for the meta-analysis of the number of vitreoretinal reoperations due to postoperative PVR. **(A)** Overall meta-analysis; **(B)** Subgroup analysis according to preoperative PVRC ratio.

**Figure 8:**
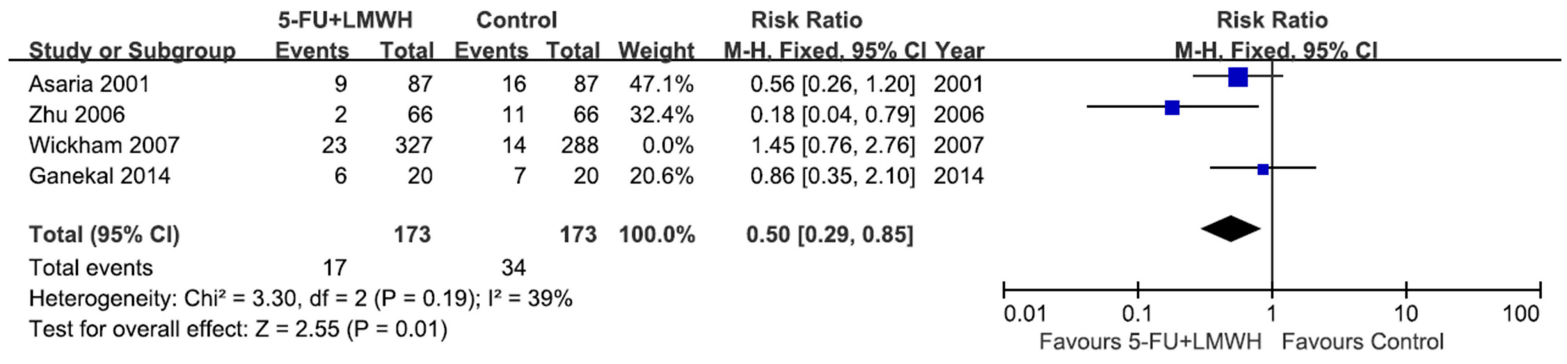
Forest plot for the meta-analysis of the number of vitreoretinal reoperations due to postoperative PVR when the study Wickham 2007 was omitted.

This outcome was reported in four studies (16, 18, 24, 29). Our meta-analysis demonstrated that 5-FU and LMWH had no effect on reducing the number of vitreoretinal reoperations due to postoperative PVR (pooled RR = 0.71, 95% CI = 0.34, 1.45, *P* = 0.34, *I*^2^ = 63%) (Figure 7A). However, in the subgroup analysis, the treatment efficacy was significant in the subgroup of patients with PVRC before intervention (*P* = 0.02, favors 5-FU and LMWH) (Figure 7B). Nevertheless, there was only one study included in this subgroup. Other subgroup analyses neither reduced heterogeneity nor changed the overall effect of 5-FU and LMWH (Supplementary Figure 4). In the sensitivity analysis, the omission of Zhu (2006) (29) or Wickham (2007) (18) decreased the heterogeneity. In addition, when Wickham (2007) (18) was omitted, the overall effect of the treatment changed (*P* = 0.01). A significant effect of combined 5-FU and LMWH on reducing the number of vitreoretinal reoperations due to postoperative PVR was observed in the remaining 3 studies (Table 6, Figure 8).

**Table 6:** Sensitivity analysis for additional outcome: number of vitreoretinal reoperations due to postoperative PVR.

**Omitted study**	**RR**	**95% CI**		**P of chi-square**	***I*** **^2^**	**Overall effect**	**Selected model**
None	0.71	0.34	1.45	0.05	63%	*P* = 0.34	random-effect model
Asaria et al. (16)	0.73	0.27	2	0.04	70%	*P* = 0.54	random-effect model
Zhu et al. (29)	0.96	0.63	1.48	0.17	43%*	*P* = 0.87	fixed-effect model
Wickham et al. (18)	0.5	0.29	0.85	0.19	39%*	*P* = 0.01**	fixed-effect model
Ganekal and Dorairaj (24)	0.62	0.22	1.74	0.02	75%	*P* = 0.37	random-effect model

* *Heterogeneity decreased after a particular study was omitted*.

** *Overall effect changed when a particular study was omitted*.

#### Visual Acuity

Methods of describing visual acuity (VA) varied among studies. Two studies classified the change of VA into “no change, better or worse” (16, 25), two sorted the VA before and after surgery into several extents (for example, CF to 0.02, 0.03–0.09, 0.1–0.2) (24, 29), and the other two presented the mean logMAR VA with or without the interquartile range (17, 18). In general, no significant difference in VA change was reported between 5-FU and LWMH group and control group (Supplementary Table 1). It should be noted that one study reported a significant decrease in VA in the 5-FU and LMWH treatment groups in macular-sparing RD patients, and authors ascribed this result to the retinal toxicity of 5-FU (18).

#### Complications

Perioperative and postoperative complications were reported in four studies (16–18, 24). No specific complications were ascribed to the combined use of 5-FU and LMWH, as summarized in Supplementary Table 2, except one study (18) that had suspected the retinal toxicity of the treatment, as mentioned above.

### Publication Bias

**Figure 9:**
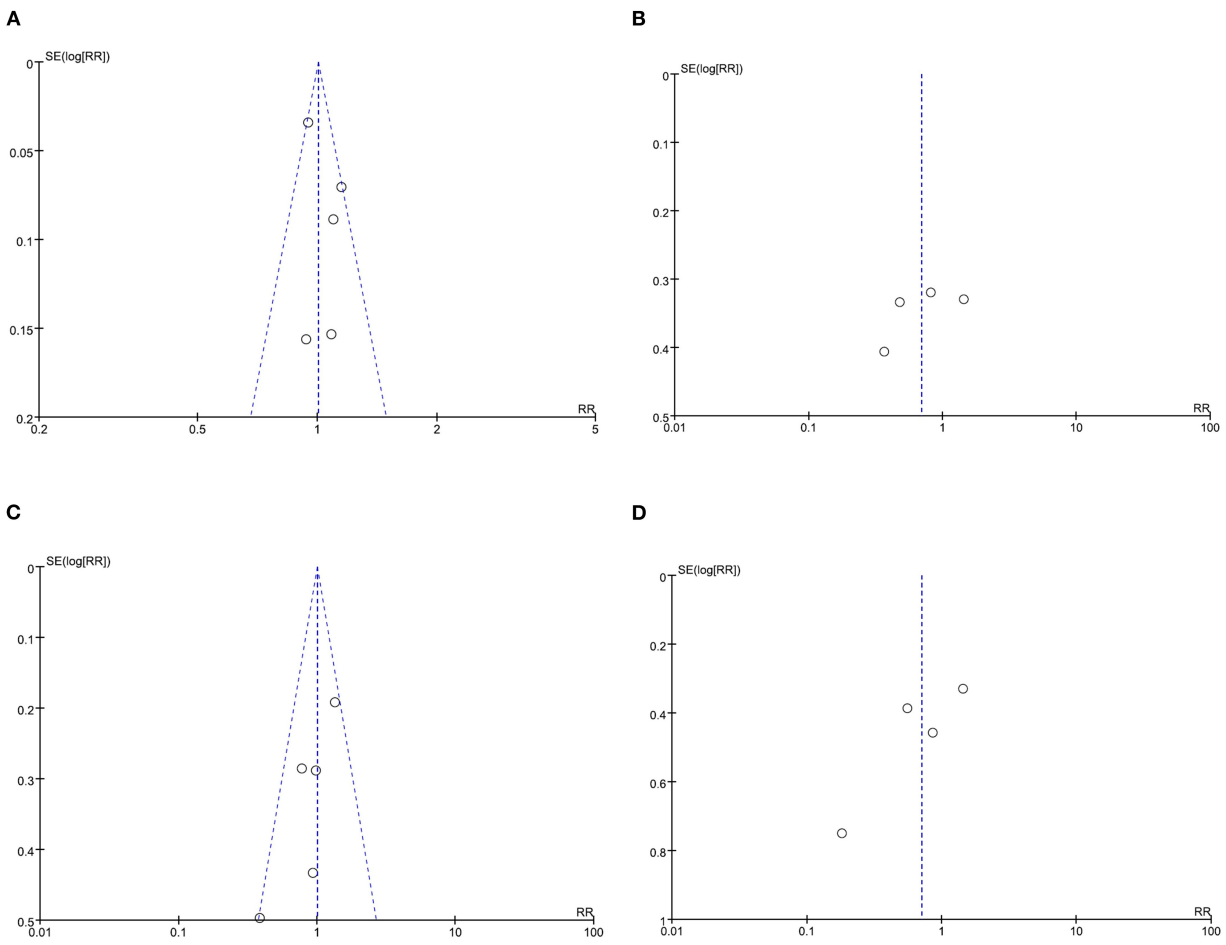
Funnel plots demonstrate the publication bias. **(A)** Funnel plot for the outcome primary success at 6 months; **(B)** Funnel plot for the outcome postoperative PVR occurrence; **(C)** Funnel plot for the outcome number of patients who underwent vitreoretinal reoperations; **(D)** Funnel plot for the outcome number of vitreoretinal reoperations due to postoperative PVR.

Funnel plots of the meta-analyses for all four outcomes were shown in Figure 9. The plots were symmetric on visual inspection, indicative of small publication bias.

## Discussion

The formation of PVR after retinal reattachment surgery is a frustrating event to retinal surgeons. In the past two decades, various drugs including steroids (32), methotrexate (8, 33), isotretinoin (7), and anti-VEGF reagents (9) have been tested in clinical trials to prevent postoperative PVR. However, none of them was routinely used in patients with RD due to the uncertainty of efficacy and possible retinal toxicity. In the current meta-analysis, we integrated data from four RCTs and two NRSI. We found that combined intraoperative infusion of 5-FU and LMWH neither increased primary success of vitrectomy at 6 months nor reduced the number of patients that underwent vitreoretinal reoperations. Subgroup analyses revealed the preoperative PVRC ratio to be the major source of heterogeneity. However, in none of these subgroups, the overall effect of the conjunct therapy was significant. Sensitivity analyses demonstrated Zhu (2006) (29) and Wickham (2007) (18) to be the potential source of heterogeneity. Nevertheless, the omission of either study did not change the overall conclusion, indicating that our meta-analyses for these two outcomes were quite reliable.

On the other hand, concerning whether the 5-FU and LMWH treatment reduced postoperative PVR, or decreased the number of vitreoretinal reoperations due to postoperative PVR, our meta-analyses were not stable. For both outcomes, in the subgroup of patients with PVRC before the intervention, the results significantly favored the conjunct therapy group. We have to take this result cautiously because there was only one study (Zhu 2006) in this subgroup. Moreover, in the sensitivity analyses for these two outcomes, the omission of Wickham (2007) (18) changed the overall conclusion and favored the conjunct therapy. Wickham (2007) (18) was a well-designed high-quality RCT. Nevertheless, it was the only study that included unselected RRD patients, with the preoperative PVRC ratio of 1.8% (treatment) and 3.7% (control), which was far lower than other included studies. This may account for the different effects of the conjunct therapy in this RCT compared with other studies. It is noteworthy that the determination of PVR is subjective, and the judgment may vary among observers. In studies where concealment was not performed (24, 25, 29), the risk of bias in the measurement of postoperative PVR occurrence would be relatively high. In our meta-analyses, when Wickham (2007) (18) was omitted, pooled data from the three remaining studies (16, 24, 29) indicated that the 5-FU and LMWH treatment reduced the postoperative PVR and decreased the number of reoperations caused by postoperative PVR in patients with a high risk of PVR and patients with PVRC before intervention. However, more studies are needed in the future to verify these results. The preoperative risk factors associated with the failure of vitrectomy for RRD include choroidal detachment, hypotony, four detached quadrants, giant retinal breaks (34), previous lens extraction, and preoperative PVRC (26). On the other hand, the commonest cause of failure of vitrectomy for RRD includes missed retinal breaks and postoperative PVR (35). It is possible that although postoperative PVR was inhibited by 5-FU and LMWH treatment in certain participants, the overall primary success of vitrectomy for RRD was not improved, as PVR occurrence was not the only cause of failure of vitrectomy.

In the literature search process, we noticed a registered study at clinicaltrial.gov (NCT 02834559). The protocol of this study was published in 2018 (36). This was a multicenter RCT approaching the effect of 5-FU and LMWH treatment on the postoperative occurrence of PVRC in primary RRD patients at high risk of PVR. Instead of subjective judgment of the grade of preoperative PVR, this study assessed PVR risk by aqueous flare measurement using laser flare photometry (36). The study results have not been published yet. We contacted the principal investigator, Dr. Schaub, and were told that the study data were still under evaluation. However, in a recent publication, Dr. Schaub implied that 5-FU and LWMH therapy may not be beneficial in this registered trial (37). The details of this study in later publications will add up to our understanding in this field.

Eyes with retinal detachment may retain fair visual acuity after retinal reattachment surgery, especially when the macular is spared. One of our included studies (18) reported a significant reduction of VA in the 5-FU and LMWH treatment group in macular-sparing RD. This was not seen in the other five studies (16, 17, 24, 25, 29) probably because these studies included patients at high risk of PVR and patients with PVRC before intervention. The general poor VA before and after surgery in these patients might mask the possible harmful effect of the conjunct therapy on the retina. LMWH has not been reported to have toxicity to the retina. However, a high dose of 5-FU showed retinal toxicity in animal models. Stern et al. reported that injection of 1.25 mg fluorouracil every 12 h for 4 days and then every 24 h for 3 days had caused an irreversible decrease in electroretinographic b-wave in vitrectomized New Zealand albino rabbits, while injection of 0.5mg fluorouracil daily for 7 days was well-tolerated in the rabbit eye (38). In all our included studies, the 5-FU infusion concentration was 200 μg/ml, which was lower than the harmless dose in animal experiments (assume the eyeball volume of rabbits to be 2.2 ml, 0.5 mg in the eyeball would be 227 μg/ml). Also, the exposure time in all the included studies was far shorter compared with 7 days. Theoretically, 200 μg/ml of 5-FU infusion was a safe dose. However, animal studies may not be comparable to clinical trials. Moreover, in vitrectomy for RRD patients, the 5-FU and LMWH infusion not only works in the vitreous cavity as in animal studies but also goes into the subretinal space through the retina tear and acts directly on the RPE and photoreceptor outer segments, which may cause extra injury to the retina. Based on all these pieces of evidence, the 5-FU and LMWH intraoperative infusion should not be used in RD patients with potential good VA.

Our meta-analysis has limitations. First, the three high-quality RCTs were conducted fully or partially in the same center (Moorfields eye hospital, London), and this would bring about selection bias. Second, the limited number of studies and the difference in participants contributed to the unstableness of the meta-analyses for two outcomes (postoperative PVR occurrence, and a number of vitreoretinal reoperations due to PVR). The positive results (*P* < 0.05) from a one-study subgroup or after a particular study was omitted need to be taken with caution. More studies in patients at high risk of PVR and patients with PVRC before intervention are needed to confirm these results.

In conclusion, our study demonstrated that the intraoperative infusion of combined 5-FU and LMWH was not effective in improving the primary success of vitrectomy for RRD or in reducing the number of patients who underwent vitreoretinal reoperations. The treatment proved beneficial in reducing postoperative PVR in patients with PVRC before the intervention, however, more studies are needed to confirm this result. Based on current evidence, 5-FU and LMWH therapy should not be routinely used in vitrectomy for RRD patients, especially in patients with potential good VA.

## Data Availability Statement

The original contributions presented in the study are included in the article/Supplementary Material, further inquiries can be directed to the corresponding author/s.

## Author Contributions

CC conceived the study, performed literature search, quality assessment, data extraction and meta-analysis, and participated in paper writing. PC performed the literature search, quality assessment, data extraction, and participated in meta-analysis and paper writing. XL participated in quality assessment and paper writing. HL conceived the study, interpreted the data, wrote the paper, and helped in quality assessment and data extraction. All authors contributed to the article and approved the submitted version.

## Funding

This study was supported by the National Natural Science Foundation of China (No. 81660167), Personnel project funded by the Health Commission of Yunnan Province (H-2019057), and Research Grant YXZX-2019013 from the Yunnan Clinical Medicine Center for Ocular Disease.

## Conflict of Interest

The authors declare that the research was conducted in the absence of any commercial or financial relationships that could be construed as a potential conflict of interest.

## Publisher's Note

All claims expressed in this article are solely those of the authors and do not necessarily represent those of their affiliated organizations, or those of the publisher, the editors and the reviewers. Any product that may be evaluated in this article, or claim that may be made by its manufacturer, is not guaranteed or endorsed by the publisher.

## References

[1] Kwon OW Song JH Roh MI. Retinal detachment and proliferative vitreoretinopathy. Dev Ophthalmol. (2016) 55:154–62. 10.1159/000438972 26501375

[2] van Leeuwen R Haarman AEG van de Put MAJ Klaver CCW Los LI Dutch G. Rhegmatogenous retinal detachment study, association of rhegmatogenous retinal detachment incidence with myopia prevalence in the Netherlands. JAMA Ophthalmol. (2021) 139:85–92. 10.1001/jamaophthalmol.2020.5114 33237293 PMC7689575

[3] Soni C Hainsworth DP Almony A. Surgical management of rhegmatogenous retinal detachment: a meta-analysis of randomized controlled trials. Ophthalmology. (2013) 120:1440–7. 10.1016/j.ophtha.2012.12.033 23511114

[4] Steel D. Retinal detachment. BMJ Clin Evid. (2014) 2014:0710. PMC3940167 24807890

[5] Idrees S Sridhar J Kuriyan AE. Proliferative vitreoretinopathy: a review. Int Ophthalmol Clin. (2019) 59:221–40. 10.1097/IIO.0000000000000258 30585928 PMC6310037

[6] Pastor JC Rojas J Pastor-Idoate S Di Lauro S Gonzalez-Buendia L Delgado-Tirado S. Proliferative vitreoretinopathy: a new concept of disease pathogenesis and practical consequences. Prog Retin Eye Res. (2016) 51:125–55. 10.1016/j.preteyeres.2015.07.005 26209346

[7] London NJS Kaiser RS Khan MA Alshareef RA Khuthaila M Shahlaee A . Determining the effect of low-dose isotretinoin on proliferative vitreoretinopathy: the DELIVER trial. Br J Ophthalmol. (2019) 103:1306–13. 10.1136/bjophthalmol-2018-312839 30381390

[8] Roca JA Yon-Mendoza A Huaman N Wu L. Adjunctive serial post-operative intravitreal methotrexate injections in the management of advanced proliferative vitreoretinopathy. Graefes Arch Clin Exp Ophthalmol. (2021) 259:2913–7. 10.1007/s00417-021-05206-z 33900444

[9] Tousi A Hasanpour H Soheilian M. Intravitreal injection of bevacizumab in primary vitrectomy to decrease the rate of retinal redetachment: a randomized pilot study. J Ophthalmic Vis Res. (2016) 11:271–6. 10.4103/2008-322X.188390 27621784 PMC5000529

[10] Goirand F Lemaitre F Launay M Tron C Chatelut E Boyer JC . How can we best monitor 5-FU administration to maximize benefit to risk ratio? Expert Opin Drug Metab Toxicol. (2018) 14:1303–13. 10.1080/17425255.2018.1550484 30451549

[11] Green E Wilkins M Bunce C Wormald R. 5-Fluorouracil for glaucoma surgery. Cochrane Database Syst Rev. (2014). CD001132. 10.1002/14651858.CD001132.pub2 24554410 PMC10558100

[12] Kon CH Occleston NL Foss A Sheridan C Aylward GW Khaw PT. Effects of single, short-term exposures of human retinal pigment epithelial cells to thiotepa or 5-fluorouracil: implications for the treatment of proliferative vitreoretinopathy. Br J Ophthalmol. (1998) 82:554–60. 10.1136/bjo.82.5.554 9713065 PMC1722588

[13] Ward T Hartzer M Blumenkranz M Lin LR. A comparison of 5-fluorouridine and 5-fluorouracil in an experimental model for the treatment of vitreoretinal scarring. Curr Eye Res. (1993) 12:397–401. 10.3109/02713689309024621 8344064

[14] Blumenkranz MS Hartzer MK Iverson D. An overview of potential applications of heparin in vitreoretinal surgery. Retina. (1992) 12(3 Suppl):S71–4. 10.1097/00006982-199212031-00015 1455088

[15] Kumar A Nainiwal S Sreenivas B. Intravitreal low molecular weight heparin in PVR surgery. Indian J Ophthalmol. (2003) 51:67–70. 12701865

[16] Asaria RH Kon CH Bunce C Charteris DG Wong D Khaw PT . Adjuvant 5-fluorouracil and heparin prevents proliferative vitreoretinopathy: results from a randomized, double-blind, controlled clinical trial. Ophthalmology. (2001) 108:1179–83. 10.1016/S0161-6420(01)00589-9 11425671

[17] Charteris DG Aylward GW Wong D Groenewald C Asaria RH Bunce C . A randomized controlled trial of combined 5-fluorouracil and low-molecular-weight heparin in management of established proliferative vitreoretinopathy. Ophthalmology. (2004) 111:2240–5. 10.1016/j.ophtha.2004.05.036 15582080

[18] Wickham L Bunce C Wong D McGurn D Charteris DG. Randomized controlled trial of combined 5-Fluorouracil and low-molecular-weight heparin in the management of unselected rhegmatogenous retinal detachments undergoing primary vitrectomy. Ophthalmology. (2007) 114:698–704. 10.1016/j.ophtha.2006.08.042 17398320

[19] Sundaram V Barsam A Virgili G. Intravitreal low molecular weight heparin and 5-Fluorouracil for the prevention of proliferative vitreoretinopathy following retinal reattachment surgery. Cochrane Database Syst Rev. (2013). CD006421. 10.1002/14651858.CD006421.pub3 23440808

[20] Page MJ McKenzie JE Bossuyt PM Boutron I Hoffmann TC Mulrow CD . The PRISMA 2020 statement: an updated guideline for reporting systematic reviews. Syst Rev. (2021) 10:89. 10.1186/s13643-021-01626-4 33781348 PMC8008539

[21] Moher D Liberati A Tetzlaff J Altman DG Group P. Preferred reporting items for systematic reviews and meta-analyses: the PRISMA statement. PLoS Med. (2009) 6:e1000097. 10.1371/journal.pmed.1000097 19621072 PMC2707599

[22] Sterne JAC Savovic J Page MJ Elbers RG Blencowe NS Boutron I . RoB 2: a revised tool for assessing risk of bias in randomised trials. BMJ. (2019) 366:l4898. 10.1136/bmj.l4898 31462531

[23] Sterne JA Hernan MA Reeves BC Savovic J Berkman ND Viswanathan M . ROBINS-I: a tool for assessing risk of bias in non-randomised studies of interventions. BMJ. (2016) 355:i4919. 10.1136/bmj.i4919 27733354 PMC5062054

[24] Ganekal S Dorairaj S. Effect of intraoperative 5-fluorouracil and low molecular weight heparin on the outcome of high-risk proliferative vitreoretinopathy. Saudi J Ophthalmol. (2014) 28:257–61. 10.1016/j.sjopt.2014.03.005 25473340 PMC4250514

[25] Garcia RA Sanchez JG Arevalo JF. Combined 5-fluorouracil, low-molecular-weight heparin, and silicone oil in the management of complicated retinal detachment with proliferative vitreoretinopathy grade C. Ophthalmic Surg Lasers Imaging. (2007) 38:276–82. 10.3928/15428877-20070701-02 17674917

[26] Wickham L Bunce C Wong D Charteris DG. Retinal detachment repair by vitrectomy: simplified formulae to estimate the risk of failure. Br J Ophthalmol. (2011) 95:1239–44. 10.1136/bjo.2010.190314 21325394

[27] Wickham L Ho-Yen GO Bunce C Wong D Charteris DG. Surgical failure following primary retinal detachment surgery by vitrectomy: risk factors and functional outcomes. Br J Ophthalmol. (2011) 95:1234–8. 10.1136/bjo.2010.190306 21156702

[28] Wang Y Wang L Zhu Z Liu B Pan A Huang L. Electroretinogram of 5-fluorouracil and LMWH prevention of proliferative vitreoretinopathy. Int J Ophthalmol. (2006) 6:651–3.

[29] Zhu Z Wang L Liu B Wang Y. A clinical trial of 5-fluorouracil combined with low molecular weight Heparin for the prevention from proliferative vitreoretinopathy. J Fourth Military Med Univ. (2006) 15:1421–4.

[30] Machemer R Aaberg TM Freeman HM Irvine AR Lean JS Michels RM. An updated classification of retinal detachment with proliferative vitreoretinopathy. Am J Ophthalmol. (1991) 112:159–65. 10.1016/S0002-9394(14)76695-4 1867299

[31] The Retina Society Terminology Committee. The classification of retinal detachment with proliferative vitreoretinopathy. Ophthalmology. (1983) 90:121–5. 10.1016/S0161-6420(83)34588-7 6856248

[32] Banerjee PJ Quartilho A Bunce C Xing W Zvobgo TM Harris N . Slow-release dexamethasone in proliferative vitreoretinopathy: a prospective, randomized controlled clinical trial. Ophthalmology. (2017) 124:757–67. 10.1016/j.ophtha.2017.01.021 28237428

[33] Falavarjani KG Hadavandkhani A Parvaresh MM Modarres M Naseripour M Alemzadeh SA. Intra-silicone oil injection of methotrexate in retinal reattachment surgery for proliferative vitreoretinopathy. Ocul Immunol Inflamm. (2020) 28:513–6. 10.1080/09273948.2019.1597894 31136255

[34] Adelman RA Parnes AJ Michalewska Z Ducournau DG. European Vitreo-Retinal Society Retinal Detachment Study, clinical variables associated with failure of retinal detachment repair: the European vitreo-retinal society retinal detachment study report number 4. Ophthalmology. (2014) 121:1715–9. 10.1016/j.ophtha.2014.03.012 24766870

[35] Richardson EC Verma S Green WT Woon H Chignell AH. Primary vitrectomy for rhegmatogenous retinal detachment: an analysis of failure. Eur J Ophthalmol. (2000) 10:160–6. 10.1177/112067210001000212 10887929

[36] Schaub F Hoerster R Schiller P Felsch M Kraus D Zarrouk M . Prophylactic intravitreal 5-fluorouracil and heparin to prevent proliferative vitreoretinopathy in high-risk patients with retinal detachment: study protocol for a randomized controlled trial. Trials. (2018) 19:384. 10.1186/s13063-018-2761-x 30012187 PMC6048849

[37] Schaub F Abdullatif AM Fauser S. Proliferative vitreoretinopathy prophylaxis: mission (im)possible. Der Ophthalmol. (2021) 118:3–9. 10.1007/s00347-020-01173-8 32666172

[38] Stern WH Guerin CJ Erickson PA Lewis GP Anderson DH Fisher SK. Ocular toxicity of fluorouracil after vitrectomy. Am J Ophthalmol. (1983) 96:43–51. 10.1016/0002-9394(83)90453-1 6869479

