# Supplementary material for: Combined 5-Fluorouracil and Low Molecular Weight Heparin for the Prevention of Postoperative Proliferative Vitreoretinopathy in Patients With Retinal Detachment: A Meta-Analysis
Source: Front Med (Lausanne). 2021 Nov 30;8:790460. doi: 10.3389/fmed.2021.790460 (PMC8669826; doi:10.3389/fmed.2021.790460)
Supplement: Supplementary file 2 [file Data_Sheet_2.PDF]

Search strategy for PubMed:

(Proliferative Vitreoretinopathy OR Proliferative Vitreoretinopathies OR Vitreoretinopathies, Proliferative OR Vitreoretinopathy, Proliferative) AND (5FU OR 5-FU OR 5-Fluorouracil OR 5 Fluorouracil OR Fluorouracil) AND (Heparin, Low Molecular Weight OR LMWH OR Low Molecular Weight Heparin OR Low-Molecular-Weight Heparin OR Heparin, Low-Molecular-Weight OR Dalteparin OR Enoxaparin OR Nadroparin OR Tinzaparin)

Search strategy for Embase:

#1. 'randomized controlled trial'/exp OR 'randomized controlled trial'
#2. Randomization
#3. 'double blind procedure'
#4. 'single blind procedure'
#5. random$
#6. #1 OR #2 OR #3 OR #4 OR #5
#7. animal OR 'animal experiment'
#8. human
#9. #7 AND #8
#10. #7 NOT #9
#11. #6 NOT #10
#12. 'clinical trial'
#13. clin$ NEAR/3 trial$
#14. (singl$ OR doubl$ OR trebl$ OR tripl$) NEAR/3 (blind$ OR mask$)
#15. Placebo
#16. placebo$
#17. random$
#18. 'experimental design'
#19. 'crossover procedure'
#20. 'control group'
#21. 'latin square design'
#22. #12 OR #13 OR #14 OR #15 OR #16 OR #17 OR #18 OR #19 OR #20 OR #21
#23. #22 NOT #10
#24. #23 NOT #11
#25. 'comparative study'
#26. 'evaluation study'
#27. 'prospective study'
#28. control$ OR prospective$ OR volunteer$
#29. #25 OR #26 OR #27 OR #28
#30. #29 NOT #10
#31. #30 NOT (#11 OR #23)
#32. #11 OR #24 OR #31

#33. 'proliferative vitreoretinopathy'
#34. 'proliferative vitreoretinopathies'
#35. vitreoretinopathies, AND proliferative
#36. vitreoretinopathy, AND proliferative
#37. Vitreoretinopathy
#38. #33 OR #34 OR #35 OR #36 OR #37
#39. 5fu
#40. '5-fu'
#41. '5-fluorouracil'
#42. '5 fluorouracil'
#43. fluorouracil
#44. #39 OR #40 OR #41 OR #42 OR #43
#45. heparin, AND 'low molecular weight'
#46. lmwh
#47. 'low molecular weight heparin'
#48. 'low-molecular-weight heparin'
#49. heparin, AND 'low-molecular-weight'
#50. dalteparin
#51. enoxaparin
#52. nadroparin
#53. tinzaparin
#54. #45 OR #46 OR #47 OR #48 OR #49 OR #50 OR #51 OR #52 OR #53
#55. #38 AND #44 AND #54
#56. #32 AND #55

Search strategy for the Cochrane library:

#1 "Proliferative Vitreoretinopathy"
#2 "Proliferative Vitreoretinopathies"
#3 Vitreoretinopathies, Proliferative
#4 Vitreoretinopathy, Proliferative
#5 #1 OR #2 OR #3 OR #4
#6 5FU
#7 "5-FU"
#8 "5-Fluorouracil"
#9 "5 Fluorouracil"
#10 Fluorouracil
#11 #6 OR #7 OR #8 OR #9 OR #10
#12 Heparin, Low Molecular Weight
#13 LMWH
#14 "Low Molecular Weight Heparin"
#15 "Low-Molecular-Weight Heparin"
#16 Heparin, Low-Molecular-Weight

#17 Dalteparin
#18 Enoxaparin
#19 Nadroparin
#20 Tinzaparin
#21 #12 OR #13 OR #14 OR #15 OR #16 OR #17 OR #18 OR #19 OR #20
#22 #5 AND #11 AND #21

Search strategy for clinical trials.gov

In the box of “condition or disease”: Proliferative Vitreoretinopathy
In the box of “other terms”: Heparin

Search strategy for WHO ICTRP: Proliferative Vitreoretinopathy and Heparin

Search strategy for ISRCTN: Proliferative Vitreoretinopathy and Heparin

Search strategy for CNKI：
高级检索：
氟尿嘧啶（摘要） 并且 肝素（摘要） 并且 PVR（摘要）
