# Supplementary material for: Combined 5-Fluorouracil and Low Molecular Weight Heparin for the Prevention of Postoperative Proliferative Vitreoretinopathy in Patients With Retinal Detachment: A Meta-Analysis
Source: Front Med (Lausanne). 2021 Nov 30;8:790460. doi: 10.3389/fmed.2021.790460 (PMC8669826; doi:10.3389/fmed.2021.790460)
Supplement: Supplementary file 3 [file Data_Sheet_3.PDF]

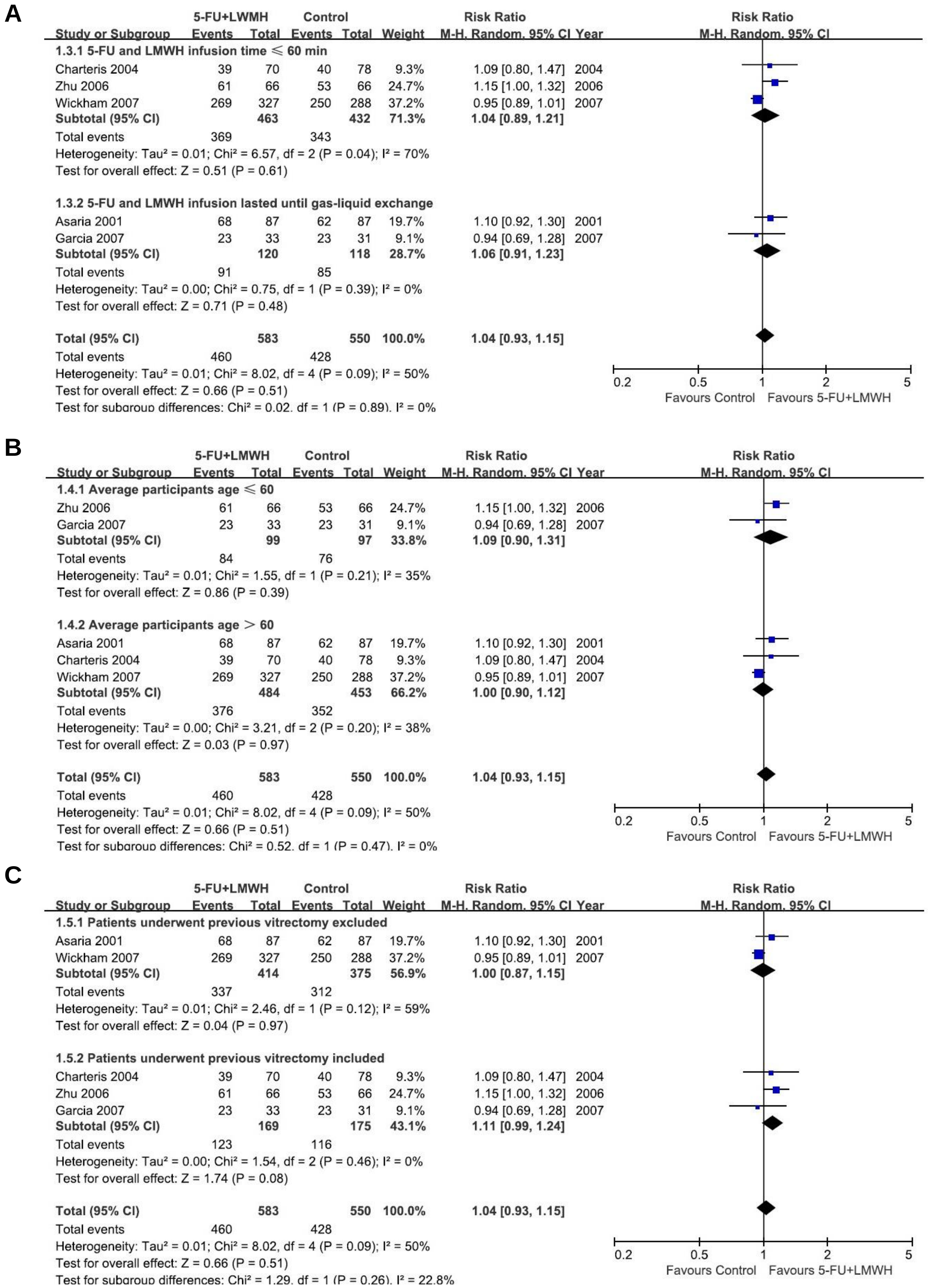

Supplementary Figure 1

Supplementary Figure 1 Forest plots for the meta-analysis of primary success at 6 months. (A) subgroup analysis according to 5-FU and LMWH infusion time; (B) subgroup analysis according to average age of participants; (C) subgroup analysis according to inclusion or exclusion of patients underwent previous vitrectomy.

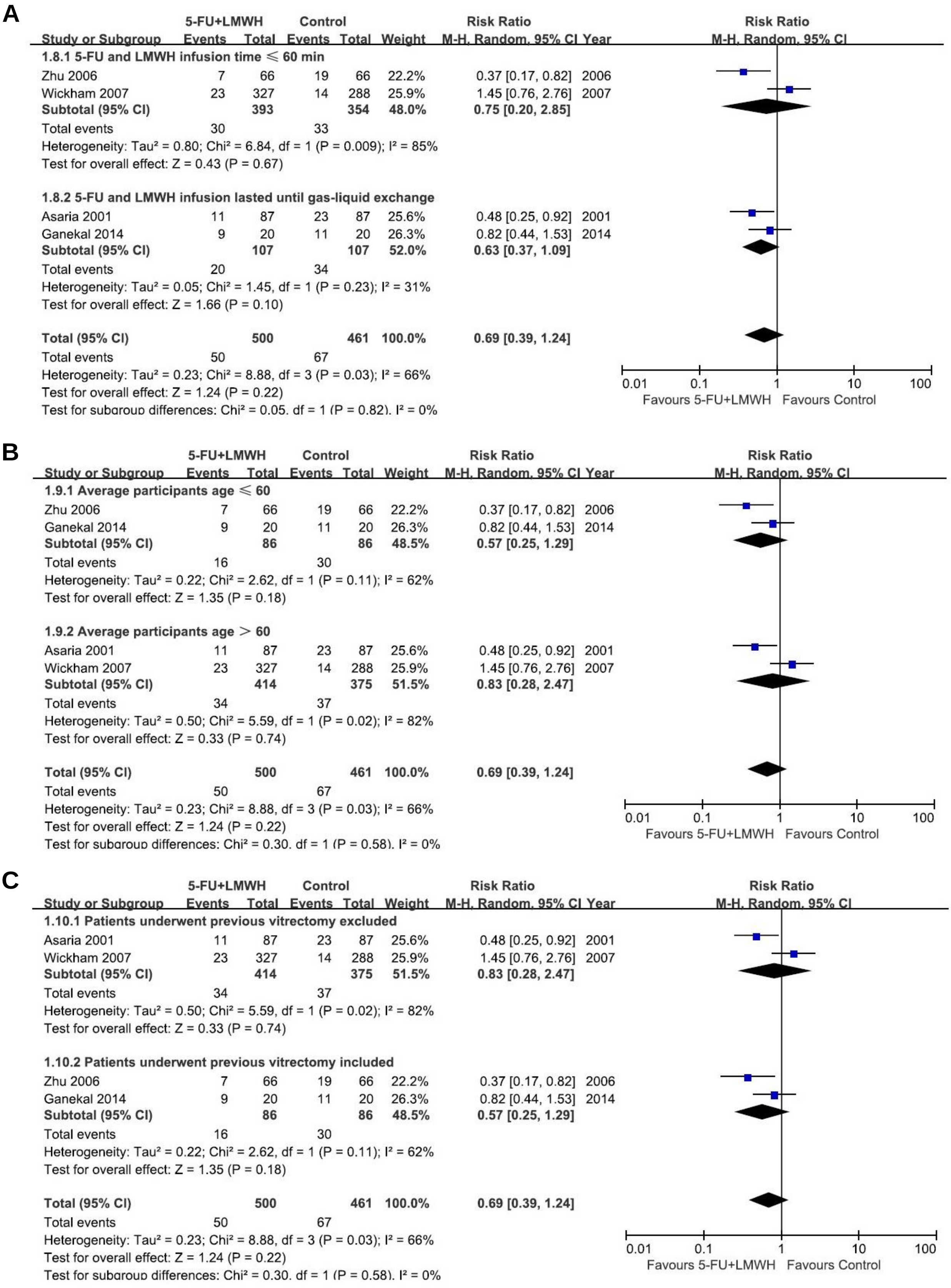

Supplementary Figure 2

Supplementary Figure 2 Forest plots for the meta-analysis of postoperative PVR occurrence. (A) subgroup analysis according to 5-FU and LMWH infusion time; (B) subgroup analysis according to average age of participants; (C) subgroup analysis according to inclusion or exclusion of patients underwent previous vitrectomy.

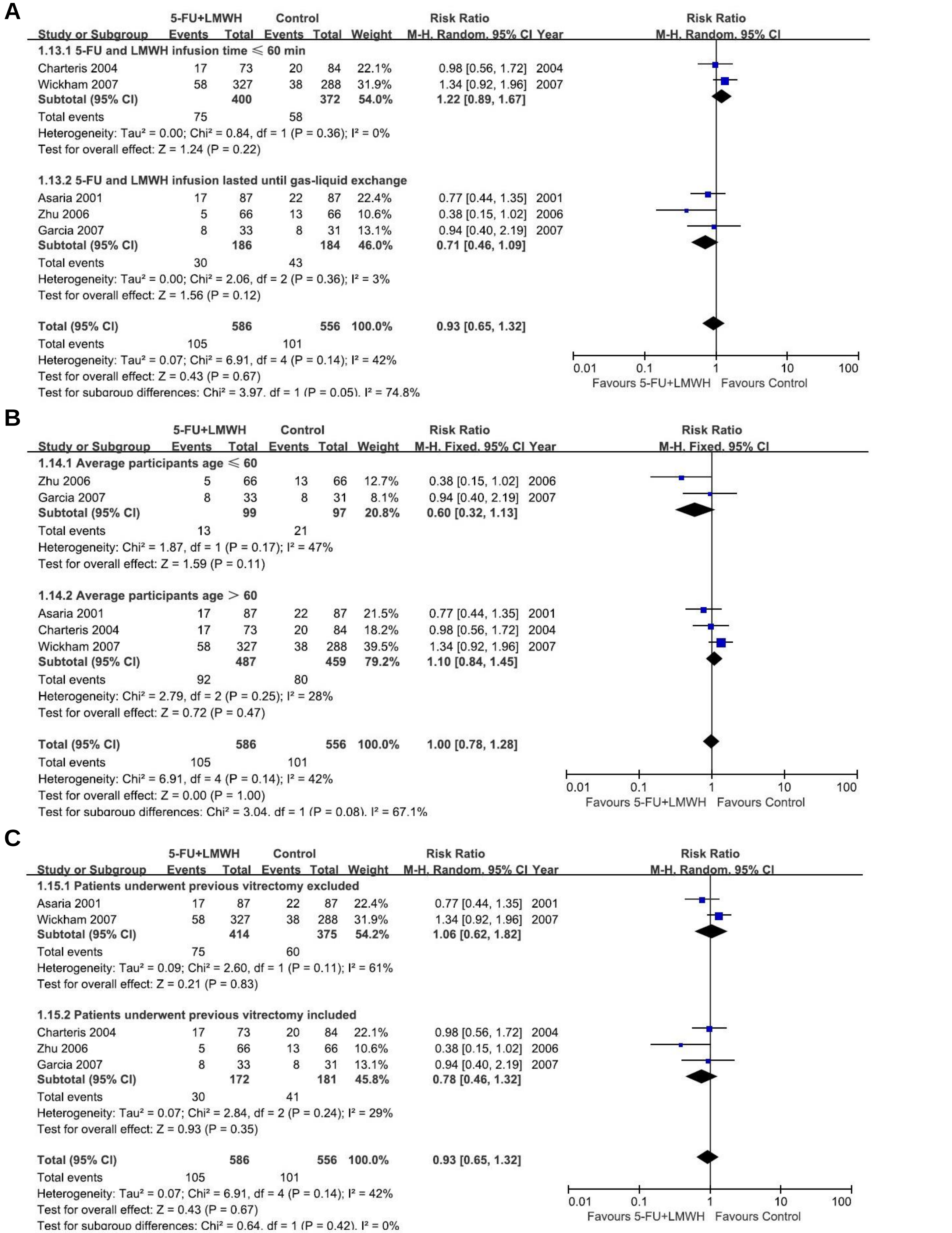

Supplementary Figure 3

Supplementary Figure 3 Forest plots for the meta-analysis of number of patients underwent vitreoretinal reoperations. (A) subgroup analysis according to 5-FU and LMWH infusion time; (B) subgroup analysis according to average age of participants; (C) subgroup analysis according to inclusion or exclusion of patients underwent previous vitrectomy.

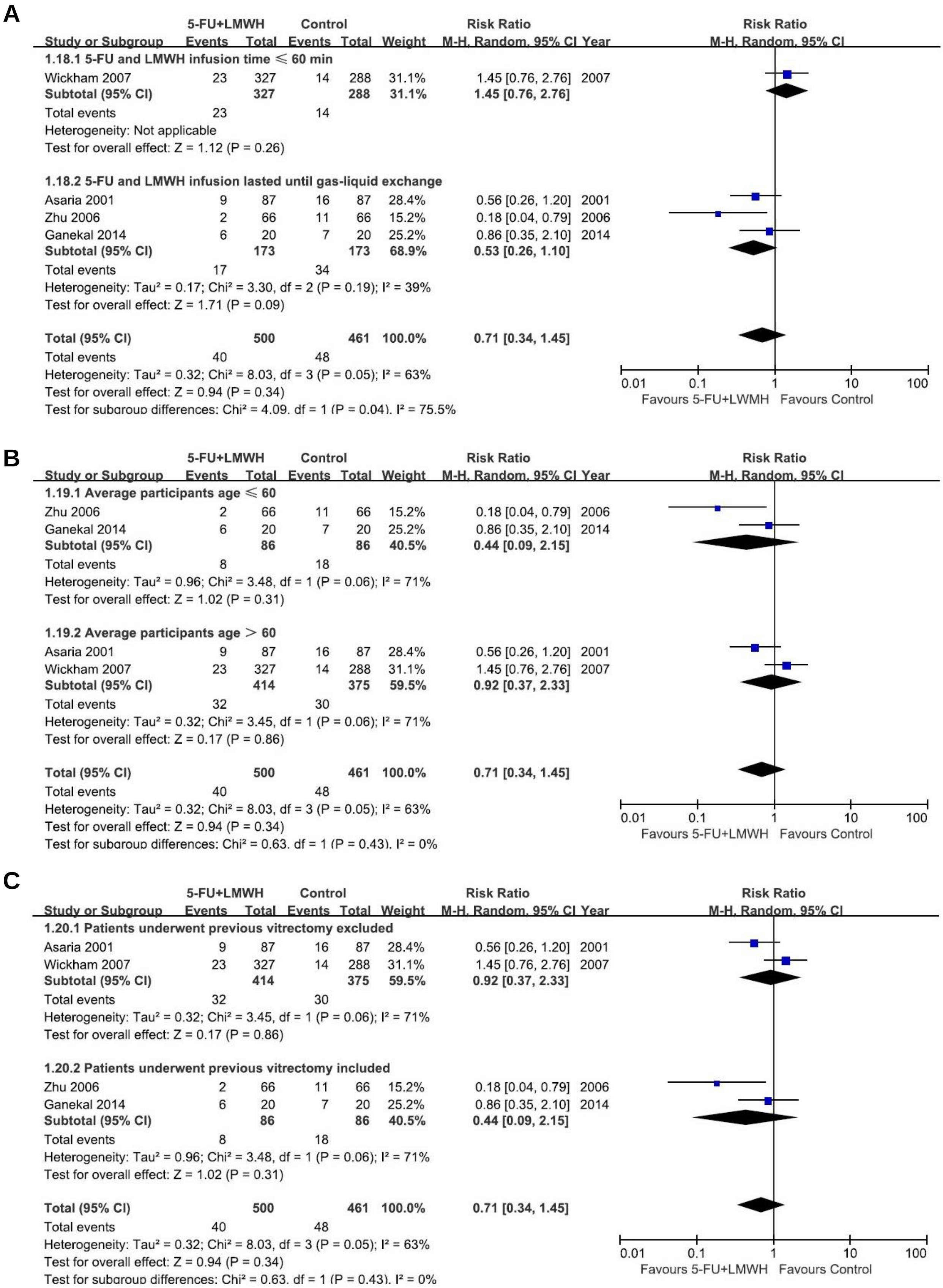

Supplementary Figure 4

Supplementary Figure 4 Forest plots for the meta-analysis of number of vitreoretinal reoperations due to postoperative PVR. (A) subgroup analysis according to 5-FU and LMWH infusion time; (B) subgroup analysis according to average age of participants; (C) subgroup analysis according to inclusion or exclusion of patients underwent previous vitrectomy.
