# Supplementary material for: Combined 5-Fluorouracil and Low Molecular Weight Heparin for the Prevention of Postoperative Proliferative Vitreoretinopathy in Patients With Retinal Detachment: A Meta-Analysis
Source: Front Med (Lausanne). 2021 Nov 30;8:790460. doi: 10.3389/fmed.2021.790460 (PMC8669826; doi:10.3389/fmed.2021.790460)
Supplement: Supplementary file 4 [file Data_Sheet_4.PDF]

Supplementary table 1. Visual acuity (VA)

Trial	VA measurement	VA result at 6 months (Treatment/Control)	Statistically Significant between treatment and control groups	Comparison
Asaria 2001	BCVA was measured using the Snellen acuity chart. Change in Visual Acuity (6 months) was described as "no change, better or worse". The difference in visual acuity was not statistically different in the two treatment groups.	No change (12/11)<br>Better (53/40)<br>Worse (22/36)	No	6 m compare with preoperative
Charteris 2004	Median logMAR VA was 2.4 in both treatment and control groups before surgery and improved to 1.8 in the treatment group and 1.4 in the control group at 6 months after surgery. The difference between the groups was not significant (P=0.126, 2-sample Wilcoxon rank-sum test).		No	6 m compare with preoperative
Wickham 2007	VA was described as best-corrected logarithm of the minimum angle of resolution visual acuity. There was no significant difference in the median final visual acuity between the treatment group (0.40; interquartile range [IQR], 0.22– 0.70) and the placebo group (0.40; IQR 0.20–0.60; P=0.0721, 2-sample Wilcoxon rank-sum test). Statistically, a significantly worse visual outcome was observed in patients who presented with a macula-sparing detachment in the treatment group (0.30; IQR 0.20–0.52) compared with the placebo group (0.22; IQR 0.12– 0.40; P 0.0091).		No (total)<br>Yes (in macular-sparing patients)	6 m compare with preoperative
Ganekal 2014	Visual outcome at the end of 6 months was classified into "<CF close to face, 20/8000–20/2666, 20/800–20/400 and 20/400–P20/200". However, no conclusion could be made due to data missing.		Not sure (data missing)	6 m compare with preoperative
Zhu 2006	Visual outcome at the end of 6 months was classified into "LP, CF to 0.02, 0.03-0.09, 0.1-0.2".		No	1,3,6 m compare with preoperative
Garcia 2007	VA was measured with ETDRS visual acuity charts using a logarithm of the minimum angle of resolution, and was described as "no change, better, worse" compared to preoperative VA.	No change (16/9)<br>Better (13/12)<br>Worse (4/10)	No	postoperative vs preoperative

Supplementary table 2. Complications

Trial	Complicaitons (n) (treatment/control)
Asaria 2001	10 postoperative hyphaemas divided equally between the two groups. Two intraoperative complications occurred in the combined treatment group; one was a retinal incarceration and the other was a choroidal hemorrhage.
Charteris 2004	Glaucoma (0/3), Hypotony (9/7), Keratopathy (5/2), Cataract extraction (21/29)
Wickham 2007	"A significant reduction in the final postoperative visual acuity was observed in patients with macula-sparing retinal detachments after treatment with 5FU and LMWH, the median logarithm of the minimum angle of resolution acuity being 0.22 in the placebo group and 0.30 in the treatment group (P=0.0091). This may be a result of retinal toxicity of the adjunctive agents in the treatment group and is of concern."
Ganekal 2014	Bleeding (1/0), Raised IOP (2/1), Synechiae (1/0), Band shaped keratopathy (1/0)
Zhu 2006	no drug toxicity observed
Garcia 2007	not mentioned
